# Hyaluronic Acid Enhances the Mechanical Properties of Tissue-Engineered Cartilage Constructs

**DOI:** 10.1371/journal.pone.0113216

**Published:** 2014-12-01

**Authors:** Peter A. Levett, Dietmar W. Hutmacher, Jos Malda, Travis J. Klein

**Affiliations:** 1 Institute of Health and Biomedical Innovation, Queensland University of Technology, 60 Musk Avenue, Kelvin Grove, QLD 4059, Australia; 2 Department of Orthopaedics, University Medical Center Utrecht, PO Box 85500, 3508 GA Utrecht, The Netherlands; 3 Department of Equine Sciences, Utrecht University, P.O. Box 80153, 3508 TD, Utrecht, The Netherlands; University of California, San Diego, United States of America

## Abstract

There is a need for materials that are well suited for cartilage tissue engineering. Hydrogels have emerged as promising biomaterials for cartilage repair, since, like cartilage, they have high water content, and they allow cells to be encapsulated within the material in a genuinely three-dimensional microenvironment. In this study, we investigated the mechanical properties of tissue-engineered cartilage constructs using *in vitro* culture models incorporating human chondrocytes from osteoarthritis patients. We evaluated hydrogels formed from mixtures of photocrosslinkable gelatin-methacrylamide (Gel-MA) and varying concentrations (0–2%) of hyaluronic acid methacrylate (HA-MA). Initially, only small differences in the stiffness of each hydrogel existed. After 4 weeks of culture, and to a greater extent 8 weeks of culture, HA-MA had striking and concentration dependent impact on the changes in mechanical properties. For example, the initial compressive moduli of cell-laden constructs with 0 and 1% HA-MA were 29 and 41 kPa, respectively. After 8 weeks of culture, the moduli of these constructs had increased to 66 and 147 kPa respectively, representing a net improvement of 69 kPa for gels with 1% HA-MA. Similarly the equilibrium modulus, dynamic modulus, failure strength and failure strain were all improved in constructs containing HA-MA. Differences in mechanical properties did not correlate with glycosaminoglycan content, which did not vary greatly between groups, yet there were clear differences in aggrecan intensity and distribution as assessed using immunostaining. Based on the functional development with time in culture using human chondrocytes, mixtures of Gel-MA and HA-MA are promising candidates for cartilage tissue-engineering applications.

## Introduction

Articular cartilage is a load bearing tissue. In articulating joints, cartilage provides low friction surfaces for efficient movement, and effective impact absorption and load dissipation. Unfortunately, cartilage is susceptible to damage, and has a very limited capacity to heal. To address this clinical need, researchers and clinicians have developed methods to potentially regenerate or tissue-engineer new cartilage, but currently there remains a shortage of materials that are well suited for guiding effective regeneration of high quality, hyaline cartilage [1].

The mechanical properties of cartilage are crucial to its ability to withstand the compressive and shear loads to which it is routinely subjected in the joint environment. It has been noted elsewhere that the importance of mechanical properties in tissue-engineered cartilage is often overlooked [2], and has even been suggested that mechanical properties should be viewed as the most important metric for assessing the quality of tissue-engineered cartilage [2]. Studies to identify materials for cartilage tissue engineering commonly characterise the mechanical properties of cell-free scaffolds or hydrogels, but frequently do not examine how the mechanical properties change with time [3]. While the optimal mechanical properties for a cartilage scaffold or tissue engineered cartilage are not known, healthy cartilage is a common reference, which has an equilibrium compressive modulus in the order of 0.1–2 MPa, varying significantly with depth from the articular surface [4]. Implanting a construct that has matured for four weeks *in vitro* showed better integration with the surrounding cartilage compared to implanting a freshly crosslinked construct [5], which may be partially a result of the mechanical properties developed over four weeks culture protecting the construct from damage once implanted.

The mechanical properties of tissue-engineered cartilage can be considered in two distinct elements. Firstly, the biomaterial has some inherent mechanical properties, and generally the mechanical properties of the newly formed construct will be derived from these inherent properties. Secondly, over time, the mechanical properties of the construct will change, due to material degradation, production of proteins and assembly of extracellular matrix via the cell machinery, and matrix remodelling.

The first element, the initial mechanical properties, is relatively simply altered by changing the materials used, the way in which they are constructed, or the way in which they are held together. For example, thermoplastic polymers can be used to produce very stiff constructs, which can easily match the stiffness of articular cartilage [6], but fail to adequately match the low friction properties and shock absorption characteristics. Hydrogels, meanwhile, have been extensively investigated for cartilage repair, but the mechanical properties of these typically soft and highly water-swollen materials have limited their clinical impact. To overcome the limitation of the mechanical properties, strategies to enhance the mechanical properties of hydrogels have been keenly investigated. For example, double network hydrogels have been developed that have excellent compliance, high water content and remarkable failure strengths [7], however the process for producing these gels is not easily adapted for cell encapsulation and the strength is reduced upon repeated loading [8]. Very stiff and strong hydrogels can also be produced from polyvinyl alcohol, and promising short term clinical results have been reported [9], but these materials replace cartilage, rather than regenerate the original tissue. Similarly, polyethylene glycol (PEG) hydrogels formed from a high concentration (40% w/v) of low molecular weight (508 Da) have mechanical properties comparable to cartilage [4], but the potential to successfully encapsulate cells in such stiff gels remains uncertain, and exposure to low molecular weight PEG may also limit cell compatibility. Yet another approach has been to combine soft, cell compatible hydrogels with stronger materials, such as thermoplastic polymers [10], [11], to produce constructs in which the hydrogel contributes negligibly to the overall mechanical properties.

In parallel, research efforts have also been directed towards developing materials that allow an increase in the mechanical properties of tissue-engineered constructs with time, usually mediated by the deposition of extracellular matrix from embedded or invading cells. For hydrogels, one common method to allow for greater increases in mechanical properties has been to *lower* the initial stiffness of the gels, usually be lowering the concentration of polymer [12], [13]. The basic principle is that reducing the polymer concentration results in a hydrogel that is not only softer, but also has larger pores, therefore allowing more diffusion of extracellular matrix into and throughout the hydrogel, which ultimately accounts for the bulk of the mechanical strength.

Hyaluronic acid (HA) is a ubiquitous component of extracellular matrices, and performs important functions in cartilage. By binding and immobilising aggrecan, HA mediates the formation of massive aggregates of fixed negative charge that retain water and is a critical extracellular matrix (ECM) component for cartilage mechanical properties. Meanwhile, the collagen network acts to resist this swelling tendency and provides tensile strength. In this study, we evaluated hydrogels formed from mixtures of photocrosslinkable derivatives of gelatin and HA (gelatin-methacrylamide (Gel-MA) and HA-methacrylate (HA-MA), respectively). In particular, we aimed to investigate in detail the role of HA-MA in the developed mechanical properties of engineered cartilage constructs using chondrocytes from four human patients. Based on our previous studies [14], we hypothesised that HA-MA would significantly enhance the mechanical properties of cultured constructs, and that the influence would be concentration dependent.

## Materials and Methods

### Ethics statement

Ethics approval was granted from the Queensland University of Technology Human Research Ethics Committee (EC00171) and the Prince Charles Hospital Human Research Ethics Committee (EC00168) (both Brisbane, Australia), and written informed consent was obtained from the donors for use of their tissue samples in research.

### Study Design

The main aim of this study was to evaluate the importance of a hyaluronic acid derivate on mechanical property development in tissue-engineered cartilage constructs, and can be considered in two parts. The objective of the first part was to characterize the mechanical properties of cell-free hydrogel constructs after 1 and 28 days in culture, including compressive modulus, swelling ratio, and also the release profile of non-covalently bond HA from Gel-MA hydrogels. The objective of the second part was to encapsulate human chondrocytes in Gel-MA/HA-MA constructs and evaluate the impact of HA-MA on the change in mechanical properties and matrix production. For the cellular experiments, the compressive modulus and swelling ratio were tested on days 1, 28 and 56, and failure strength was measured after 63 days. ECM production was assessed on constructs cultured for 28 days.

### Macromer synthesis

Gelatin and methacrylic anhydride (MAAh) were purchased from Sigma Aldrich (St Louis, MO, USA). Hyaluronic acid was purchased from Novozymes Biopharma (Bagsvaerd, Denmark). Irgacure 2959 was purchased from BASF (Ludwigshafen, Germany). Gelatin-methacrylamide (Gel-MA) and hyaluronic acid methacrylate (HA-MA) were synthesized using protocols based on previous methods [15], [16], [17]. Unless otherwise stated, all concentrations are given as percentage weight per volume (% w/v). Gelatin was dissolved in phosphate buffered saline (PBS, Invitrogen, Carlsbad, CA, USA) at 10% and reacted with 0.6 g MAAh per gram of gelatin for 1 hour at 50°C under constant stirring. After the reaction period, excess MAAh was removed by centrifugation, and the Gel-MA was exhaustively dialysed against distilled water at 40°C. The pH of the dialysed Gel-MA solution was adjusted to 7.4, and Gel-MA was recovered by lyophilisation. HA was dissolved in distilled water at 2% and reacted with MAAh on ice for 8 hours under constant stirring. HA-MA was precipitated in an excess of cold 100% ethanol, redissolved, then exhaustively dialysed against distilled water. The pH of dialysed HA-MA was adjusted to 7.4, and HA-MA was recovered by lyophilisation. Following lyophilisation, HA-MA was washed with acetone and allowed to dry overnight in a biological safety cabinet to ensure sterility. Each polymer was dissolved separately at 10 mg/mL in deuterium oxide (Sigma) and proton nuclear magnetic resonance spectra were recorded on a Mercury 300 MHz instrument (Varian Associates Inc., CA, USA).

### Cell isolation and expansion

Human chondrocytes were isolated and expanded as described in detail elsewhere [18]. Cartilage with a macroscopically normal appearance was excised from the femoral condyles of four osteoarthritis (OA) patients that had undergone knee replacement surgery. The cartilage was diced with a scalpel and digested overnight with 0.15% collagenase type II (Worthington, NJ, USA) in high glucose Dulbecco's modified eagle medium (DMEM, Invitrogen). Chondrocytes from the four patients were kept separate for the entirety of the study, and were expanded in low-glucose DMEM with 2 mM glutamax (Invitrogen), supplemented with 10% fetal bovine serum (Lonza, Waverly Australia), 10 mM 4-(2-hydroxyethyl)-1-piperazineethanesulfonic acid (HEPES), 0.1 mM non-essential amino acids, 0.5 µg/mL amphotericin B (Fungizone), 50 U/mL penicillin G sodium, 50 µg/mL streptomycin (all Invitrogen), 0.4 mM L-proline and 0.1 mM ascorbic acid (both Sigma).

### Hydrogel formation

All hydrogels were crosslinked in a custom made Teflon mould by 15 minutes exposure to 2.6 mW/cm^2^ 365 nm light (UVP CL-1000, Upland, CA, USA). All gel precursor solutions had a total polymer concentration of 10% and contained 0.05% of the photoinitiator Irgacure 2959. Passage one chondrocytes were released from monolayer culture by 5 minutes incubation with 0.25% trypsin (Invitrogen). The cells were washed with DMEM containing FBS, then washed a further two times with serum-free DMEM. The cells were counted using a hemocytometer, and encapsulated by combining one millilitre of gel precursor solution with each 10^7^ cells and photocrosslinking. Initially gels had dimensions of approximately 4×4×2 mm. Cell-hydrogel constructs were cultured for up to 9 weeks in defined chondrogenic differentiation media (high-glucose DMEM with 2 mM glutamax (Invitrogen), 10 mM HEPES, 0.1 mM nonessential amino acids, ITS-G (100× dilution), 0.5 µg/mL amphotericin B (Fungizone), 50 U/mL penicillin G sodium, 50 µg/mL streptomycin (all Invitrogen), 1.25 mg/mL bovine serum albumin (BSA), 0.4 mM L-proline, 0.1 mM ascorbic acid, 0.1 µM dexamethasone (all Sigma) and 10 ng/mL TGF-β3 (GroPep, Adelaide, Australia)). Cell-free hydrogels were cultured in high-glucose DMEM with 1.25 mg/mL BSA, 0.5 µg/mL amphotericin B, 50 U/mL penicillin G sodium and 50 µg/mL streptomycin.

### Viability analysis

After 28 days culture, cell-laden hydrogel constructs were halved with a scalpel, incubated in a solution of 10 µg/mL fluorescein diacetate and 5 µg/mL propidium iodide (both Sigma) in PBS for 10 minutes, and imaged using a Zeiss Axio microscope. The cut face of the hydrogel construct was imaged to visualise the cell viability in the centre of the construct.

### Mechanical testing

All hydrogels were tested in an unconfined arrangement between non-porous platens while submerged in PBS at 37°C, using an Instron 5848 microtester (Instron, Melbourne, Australia). The compressive modulus was determined by compressing gels at 0.01 mm/s, and calculating the slope of the stress-strain curve between 10–15% strain. The cross-sectional area of each gel was calculated from the ratio of wet weight to height. After testing, the gels were lyophilised to determine the dry weight. The (mass) swelling ratio was determined as the ratio of wet weight to dry weight. The following procedure was used to determine the equilibrium modulus, dynamic modulus, failure strength and failure strain for each gel, using a displacement rate of 0.01 mm/s. Gels were compressed to 5% strain, held for 10 minutes, compressed to 10% strain, and held for 10 minutes. Fifty cycles of a sinusoidal waveform with an amplitude of 2% and frequency of 1 Hz was applied, centred about 10% strain. Gels were then compressed to 15% strain, held for 10 minutes, and compressed to 20% strain and held for 10 minutes. The load was released completely, and the gels were compressed until failure using a displacement rate of 0.01 mm/s. For each gel, the equilibrium modulus was determined from the slope of the residual stress-strain curve between 10 and 15% strain. The dynamic modulus was determined as the slope of the stress strain curve between 9 and 11% strain during dynamic compression. The failure force and displacement were taken as the point of a clear discontinuity of the force-displacement curve, from which failure strength and strain were calculated based on cross-sectional area and height.

### Immunofluorescence

Constructs were snap frozen in Optimal Cutting Temperature (OCT) compound (Sakura, Finetek, Tokyo, Japan) and sectioned. Sections were fixed with ice-cold acetone for 10 minutes, and washed in PBS. Antigen retrieval was performed for collagen type II, using 0.1% hyaluronidase (Sigma) for 30 minutes at room temperature. All sections were blocked with 2% bovine serum albumin (BSA, Sigma) in PBS for 1 hour at room temperature. Primary antibodies were diluted in 2% BSA in PBS and applied overnight at 4°C. Antibodies for aggrecan (969D4D11, Invitrogen, 1∶300 dilution), collagen type II (II-II6B3, Developmental Studies Hybridoma Bank (DSHB), Iowa City, IA, USA, 1∶200 dilution) and CD44 (H4C4, DSHB, 1∶200 dilution) were used. Slides were washed three times in PBS for 5 minutes each. The goat anti-mouse secondary antibody (Alexa Fluor488, Invitrogen, 1∶400 dilution) was diluted in 2% BSA in PBS containing 5 µg/mL 4,6-diamidino-2-phenylindole (DAPI, Invitrogen), and applied in the dark for 1 hour. Slides were washed a further 3 times in PBS for 5 minutes each, and after drying, mounted with Prolong Gold (Invitrogen) and imaged using a Zeiss Axio microscope. Confocal microscopy was used to image the morphology of encapsulated cells and cells at the surface of constructs. Whole constructs were incubated in PBS containing 5 µg/mL DAPI and 0.8 U/mL rhodamine phalloidin for 1 hour then washed three times in PBS. Images were captured on a Leica SP5 laser scanning confocal microscope.

### Biochemical analyses

GAG content was measured quantitatively using the dimethylmethylene blue (DMMB, Sigma) assay and a standard curve of chondroitin-6-sulfate (Sigma) and the ratio of absorbance at 525 nm to 590 nm. For HA/HA-MA retention experiments the assay was performed at pH 3.0, and for cell culture experiments pH 1.5 was used, since unsulfated GAGs bind with DMMB at a pH of 3.0 but not 1.5 [19]. For the retention experiments, cell-free gels were incubated in PBS at 37°C for up to two weeks. At each time point, four gels were removed from PBS, weighed, and digested with papain (250 µg/mL, Sigma) at 60°C overnight. GAG content was measured in cell-laden constructs after 1 and 28 days culture. The constructs weighed, lyophilised, weighed again, and digested with 0.5% hyaluronidase (Sigma) in PBS at 37°C for 48 hours, followed by digestion with proteinase K (Invitrogen) overnight at 56°C. DNA content in the digests was measured using the Quant-iT PicoGreen dsDNA assay (Invitrogen).

### EPIC-μCT

Equilibrium partitioning of an ionic contrast agent (EPIC) microcomputed tomography (μCT) was used to visualize the concentration and distribution of negative charge, as a measure for proteoglycan content in hydrogel constructs [20], [21]. This technique uses a negatively charged contrast agent to indicate the amount and distribution of fixed negative charge in the hydrogel construct. Since cartilage ECM is negatively charged, gels with a higher amount of ECM, and therefore a higher negative charge, have a lower signal strength of contrast agent. Gels were incubated in a mixture of 40% Ioxaglate (Hexabrix, Aspen, Australia) in PBS at 37°C overnight with constant mixing, and imaged using a Scanco μCT 40 scanner (Scanco Medical, Brüttisellen, Switzerland).

### Statistical analyses

All statistical analyses were performed using SPSS v20 (IBM corporation, Armonk), with a statistical significance level of 0.05. Differences between hydrogel groups (0–2% HA-MA) were determined using ANOVA. Tukey's posthoc test was used in instances where the p-value for Levene's test was greater than 0.05, and Tamhane's T2 posthoc test was used where the p-value for Levene's test was less than 0.05. Statistically significant differences from the appropriate posthoc test are indicated in Figures using Roman numerals. Independent samples t-tests were used for comparisons within groups or between days, and statistically significant differences were indicated in figures using the symbols *, # and †.

## Results

All hydrogels maintained their macroscopic appearance and shape throughout the entirety of the study, and could be easily handled with a spatula. Cell-free gels were noticeably more transparent than cell-laden gels. The opacity of gel precursor solutions increased slightly with HA-MA concentration at 37°C, and much more so at 4°C Figure S1), indicating imperfect mixing of the two polymers. The solution with 2% HA-MA was notably more viscous that all other groups, and consequently more challenging to work with. Proton nuclear magnetic resonance confirmed that gelatin and HA were successfully modified with methacrylate or methacrylamide groups (Figure S2).

### Retention of HA

**Figure 1:**
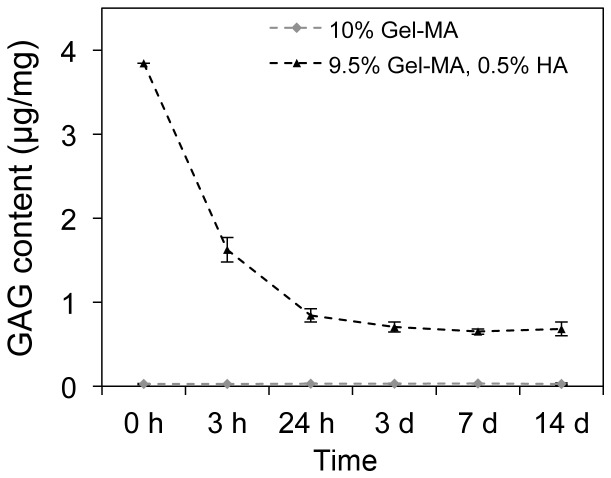
Retention of HA in cell-free Gel-MA hydrogels over 14 days. HA content per wet weight, determined using a quantitative GAG assay at a number of time points. The retention of HA-MA was not measureable, since the HA-MA appears to become crosslinked, and does not dissolve after proteinase K or papain digestion. Each point represents the mean of four samples, and error bars show standard deviations.

When unmodified HA was incorporated into Gel-MA hydrogels, a portion diffuses out of the gels over the first 1-3 days, but some HA is stably incorporated (Figure 1). HA-MA content could not be quantified using the DMMB assay after papain digestion, since after digestion of gels containing HA-MA, a soft, yet stable hydrogel remained, most probably a crosslinked network of HA-MA. This nevertheless provides reasonable evidence that HA-MA is stably incorporated, and in all studies with chondrocytes, HA-MA was therefore, used.

### Viability and cell morphology

**Figure 2:**
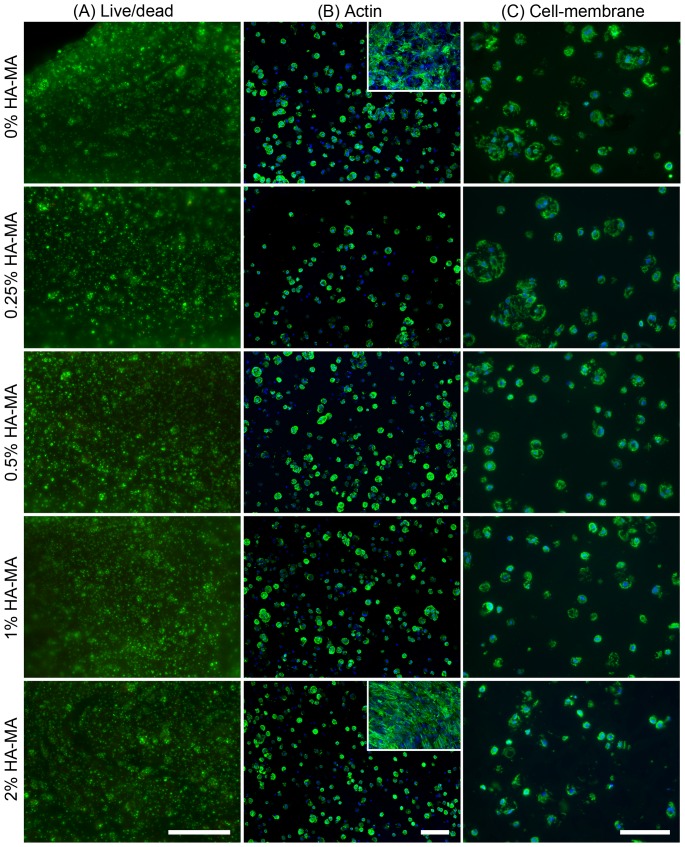
Viability and morphology of chondrocytes after 28 days culture in Gel-MA hydrogels with 0–2% HA-MA. (A) Viability images from the centre of hydrogel constructs, in which living cells appear green (stained with fluorescein diacetate) and dead cells appear red (stained with propidium iodide). (B) Confocal microscopy showing actin filaments (green) and nucluei (blue) showing the morphology of encapsulated chondrocytes inside the gels and at the surface of the gels (insets for 0 and 2% HA-MA). (C) CD44 immunostaining (green) and nuclei (blue) showing the cell membrane morphology of encapsulated cells. The scalebar in (A) represents 500 µm, and scalebars in (B) and (C) represent 100 µm.

Cell viability was not dependent of HA-MA concentration, and was high in all groups after 28 days (Figure 2A). The morphologies exhibited by cells in all gels fell into one of two major categories: encapsulated cells displayed a predominantly rounded morphology, while cells at the surface of constructs had highly spread, fibroblastic morphologies (Figure 2B and C, Figure S3). Some encapsulated cells showed slight deviations from rounded morphology, with some non-circular actin structures and cell membrane extensions observed, particularly in constructs with 0% HA-MA (Figure S3).

### Physical properties

**Figure 3:**
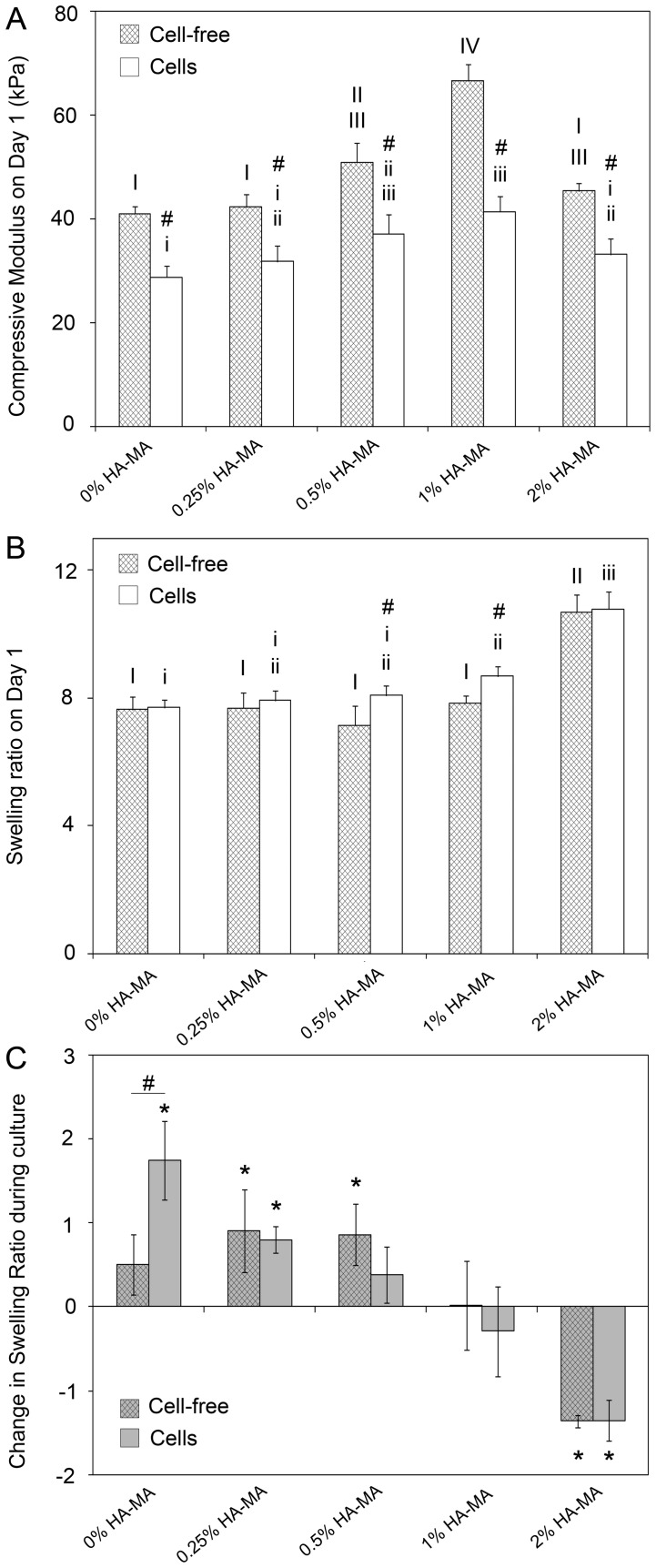
Physical properties of cell-free and cell-laden gels after 1 and 28 days culture *in vitro*. (A) Compressive modulus of cell-free and cell-laden gels on day 1; (B) swelling ratio of cell-free and cell-laden gels on day 1; and (C) change in swelling ratio between days 1 and 28 of culture. Bars and error bars represent the means and standard deviations of data from four patients. In (C), a value of 2 represents an increase in swelling ratio, for example from 8 on day 1 to 10 on day 28. The symbol # indicates a significant difference between cell-free and cell-laden gels.

On day 1, HA-MA had a relatively minor, but statistically significant impact on the compressive modulus of cell-free and cell-laden hydrogels (Figure 3A). For HA-MA concentrations up to 1%, compressive modulus increased with HA-MA concentration. Constructs with 2% HA-MA were softer than those with 1% HA-MA, which was driven by substantially greater swelling in the 2% HA-MA hydrogels (Figure 3B). On day 1, all cell-laden hydrogels were softer than their cell-free counterparts (Figure 3A), but the effect was not a consequence of differential swelling. On day 1, swelling ratios of cell-free and cell-laden gels were generally similar, with only a few small, but statistically significant differences (Figure 3B).

**Figure 4:**
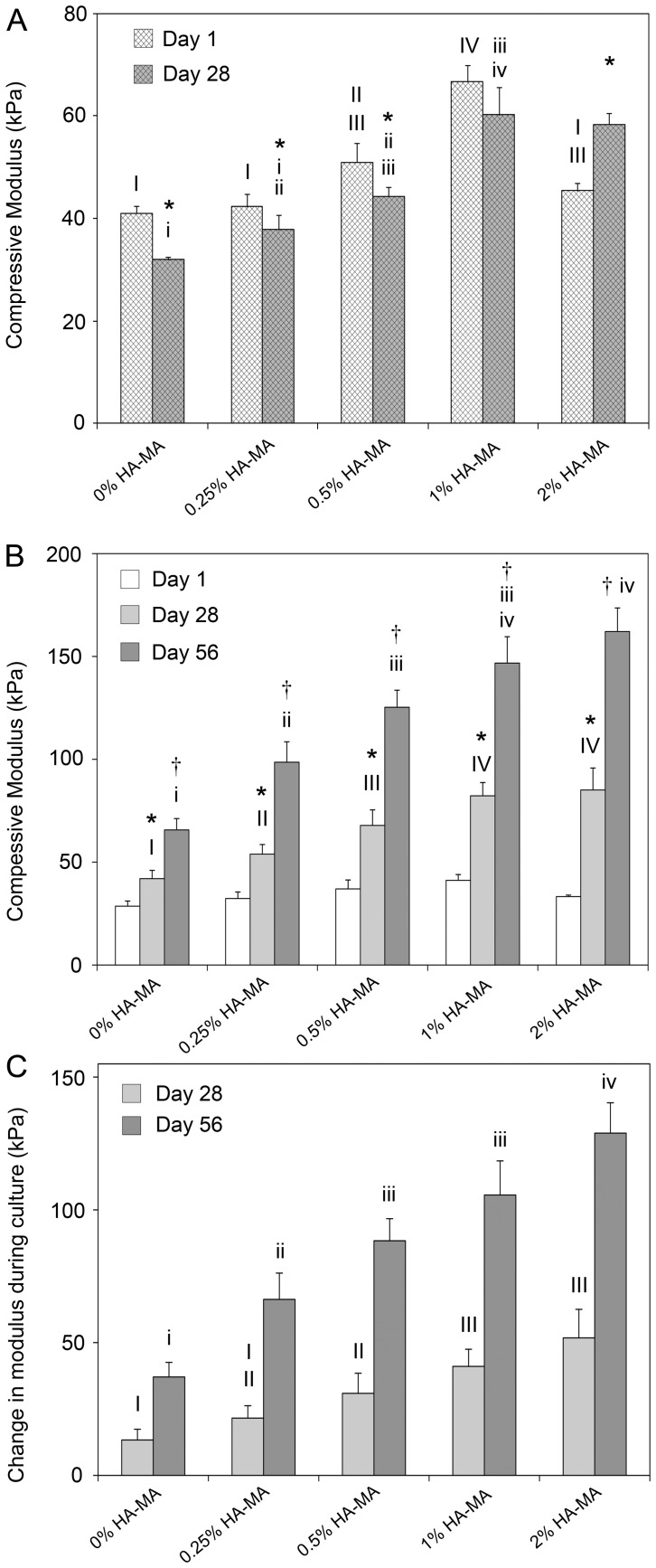
Compressive modulus of cell-free and cell-laden gels throughout *in-vitro* culture. (**A**) The compressive modulus of cell-free gels on days 1 and 28; (B) compressive modulus of cell-laden gels on days 1, 28 and 56; and (C) change in modulus between day 1 and day 28 and day 1 and day 56. The compressive modulus increased in all gels with culture time, and HA-MA had a significant impact on the rate of increase. * indicates a difference between days 1 and 28, and † indicates a difference between days 28 and 56. In each panel, groups without a like Roman numeral are significantly different, and upper and lower cases should be considered separately.

After 28 days of culture, cell-free gels with 0–0.5% HA-MA were softer than on day 1, while 1% HA-MA gels were unchanged, and 2% HA-MA increased in stiffness (Figure 4A). The increased stiffness of gels with 2% HA-MA was consistent with a notable reduction in the swelling ratio, whereas the swelling ratios of other hydrogels either increased or were unchanged (Figure 3C). For each group with HA-MA, the change in swelling ratio during culture was the same for cell-free and cell-laden gels. For the Gel-MA only group, however, the cells caused the swelling ratio to increase significantly more during culture than the cell-free gels.

The most striking effect of HA-MA was apparent for the changes in the compressive moduli of cell -laden gels during culture. HA-MA had a strong and concentration-dependent effect on the developed mechanical properties after 28 and 56 days culture (Figure 4A and B). The stiffness of all groups increased during culture, but the increases were substantially greater in constructs containing HA-MA, and generally, the change in modulus increased as HA-MA concentration increased (Figure 4C). After 56 days culture, the compressive modulus of Gel-MA only constructs (0% HA-MA) increased by an average of 37 kPa, whereas constructs with 2% HA-MA increased by an average of 129 kPa during the same period.

**Figure 5:**
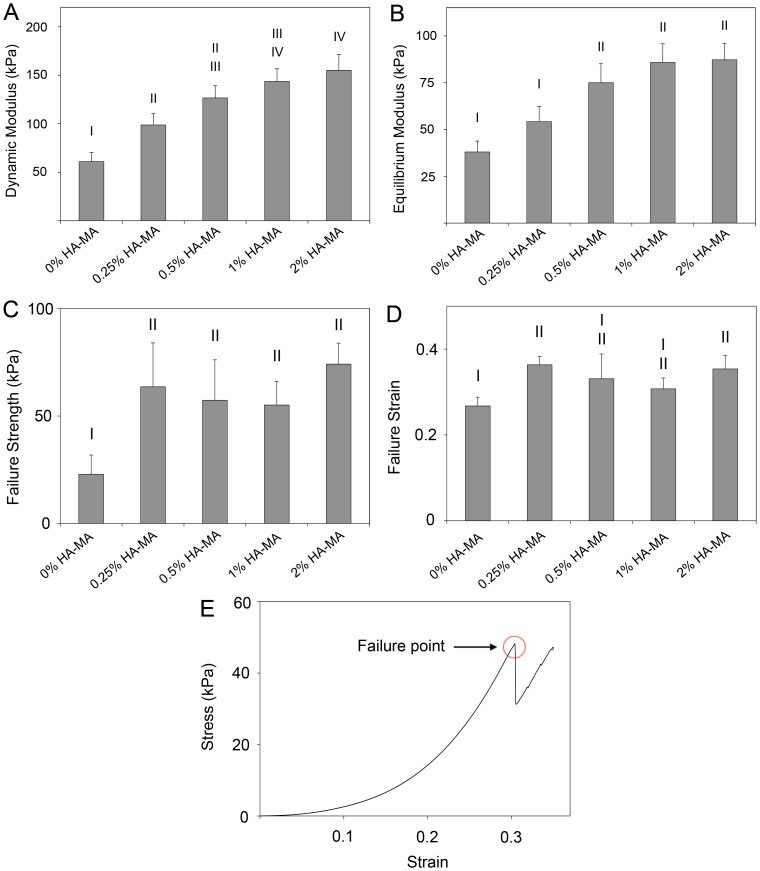
Physical properties of cell-laden gels after 63 days of culture. (A) Dynamic and (B) equilibrium moduli (B); (C) failure strength and (D) failure strain. An example stress-strain curve for a compression-to-failure test is shown in (E). Bars and error bars represent the means and standard deviations of data from four patients. Differences between groups are indicated using Roman numerals; groups with a common numeral are significantly similar, and groups without a common numeral are significantly different.

After 63 days culture, the dynamic and equilibrium moduli of cell-laden constructs were measured (Figure 5A and B). Both dynamic and equilibrium moduli tended to increase with HA-MA concentration, although for higher HA-MA concentrations, the differences were smaller and less likely to be statistically significant. The presence of clear differences in the equilibrium moduli confirms that the differences in compressive and dynamic moduli are not just a result of increased resistance to fluid flow, but also stem from increased rigidity of the network. For hydrogel materials, the compressive modulus measured at a given strain will always be equal to or higher than the equilibrium modulus at the same strain. On day 63, the equilibrium modulus of gels with 1% HA-MA was 86 kPa, significantly higher than the compressive modulus of the same gels on day 1 (41 kPa), which also confirms that a substantial component of the equilibrium stiffness can be attributed to the cell-secreted extracellular matrix.

HA-MA also had a significant impact on the failure properties of cell-laden constructs on day 63, but unlike the moduli measurements, was not strongly dependent on HA-MA concentration (Figure 5C). On average, the failure stress of gels with any concentration of HA-MA was 63 kPa, which represents a notable increase from the failure strength of gels without HA-MA (23 kPa). Failure strains followed a similar pattern to failure strength, although not all differences were statistically significant (Figure 5D).

### Matrix production

**Figure 6:**
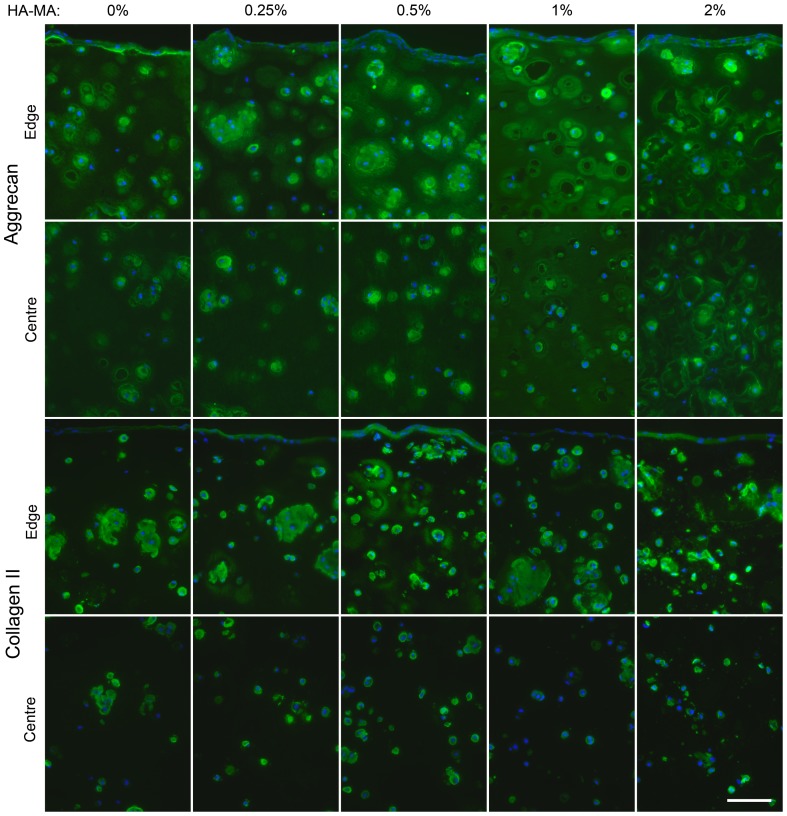
Aggrecan and collagen type II immunofluorescence in constructs after 28 days of culture. Aggrecan and collagen type II are shown in green, and nuclei are shown in blue. Regions at the edge and centre of each construct are shown. The scalebar represents 100 µm and applies to all panels.

After 4 weeks of culture, immunofluorescence was used to visualize the presence and distribution of the major cartilage matrix components aggrecan and collagen type II. For all hydrogel compositions, the intensity of both aggrecan and collagen type II immunofluorescence was greater towards the outer regions of the construct than in the centre (Figure 6). HA-MA appeared to influence the distribution of aggrecan, with more staining in the ECM as opposed to the pericellular matrix in constructs with HA-MA. Hydrogel composition appeared to have only minor impacts on the staining patterns of collagen type II, and the staining patterns of collagen type II and aggrecan were quite distinct.

**Figure 7:**
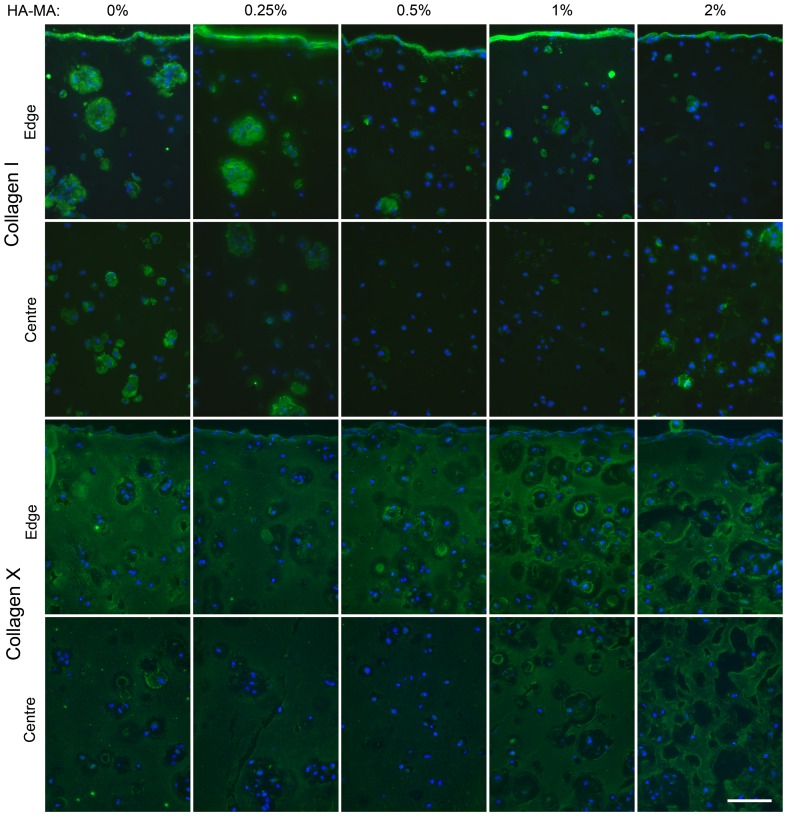
Collagen type I and type X immunofluorescence in constructs after 28 days of culture. Collagen type I and type X are shown in green and nuclei are shown in blue. Regions at the edge and centre of each construct are shown. The scalebar represents 100 µm and applies to all panels.

Collagen type 1 immunofluorescence was strong at the outermost edge of the constructs (Figure 7), consistent with the stretched chondrocyte morphologies observed on the surface of the gels. Collagen type I was also more strongly and widely stained in gels without HA-MA, especially compared to those with 0.5% and 1% HA-MA (Figure 7). Collagen type X was present throughout the cultured constructs (Figure 7), indicating that cells may be undergoing hypertrophy and potentially for the ECM to be partially mineralised.

### GAG production

**Figure 8:**
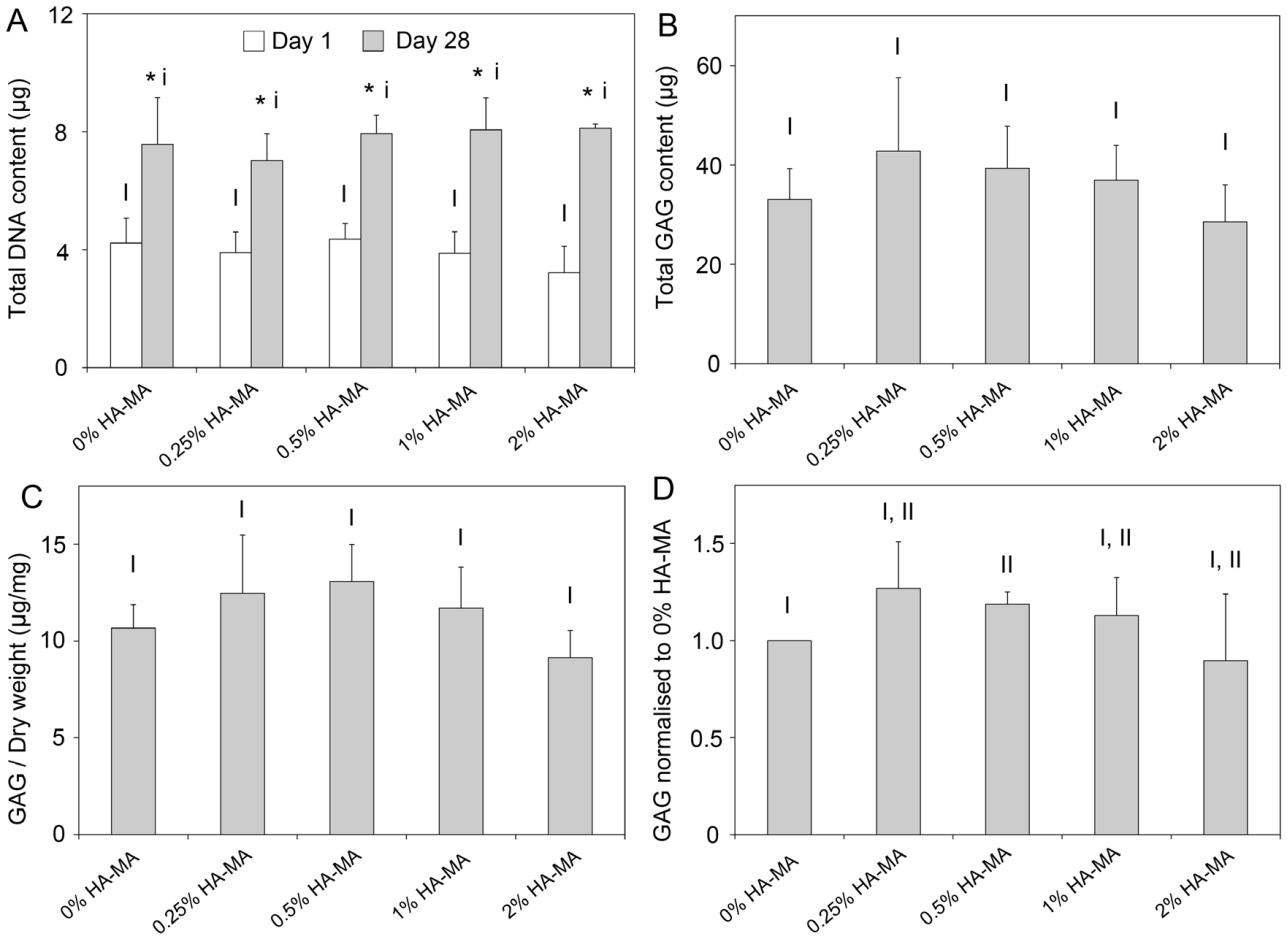
Biochemical analyses of cartilage constructs. (A) DNA content of constructs on days 1 and 28. (B) Total GAG content in each hydrogel construct after 28 days of culture; (C) GAG content normalised to dry weight, and (D) GAG content normalised to the GAG content for 0% HA-MA hydrogels for each patient (D). Bars and error bars represent the means and standard deviations of four patients. Groups without a like Roman numeral are significantly different, and those with a like numeral are statistically similar. In (A), * indicates a significant difference in the DNA content in a hydrogel group between days 1 and 28.

The amount of GAG accumulated in the constructs over 28 days culture was quantified, and DNA content was quantified on days 1 and 28. In all gels, DNA content increased by a similar amount during 28 days culture (Figure 8A). Total GAG content was statistically similar in all groups (Figure 8B), and GAG content normalised to dry weight was also similar in all groups (Figure 8C). When normalised to the total GAG content to the 0% HA-MA group for each patient to account for inter-patient variability, a modest increase is apparent in gels with 0.5% HA-MA compared to Gel-MA only gels (Figure 8D).

### EPIC-μCT

**Figure 9:**
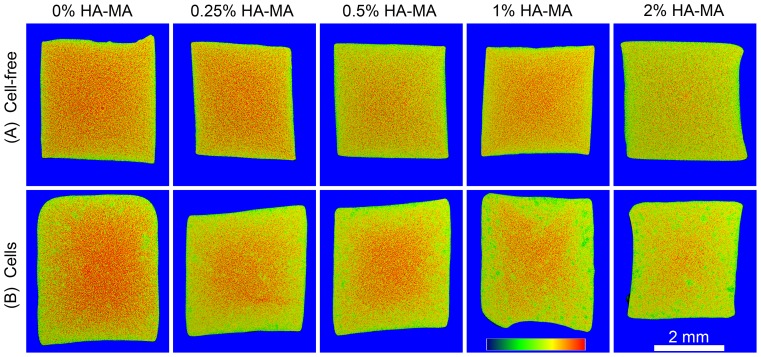
EPIC-μCT scans of cell-free (A) and cell-laden (B) constructs after 28 days culture. The scans show intensity of the negatively charged contrast agent Ioxaglate, and attenuation range in each panel is from 5,000 (blue) to 22,000 (red). The 2 mm scalebar applies to all panels.

EPIC-μCT was used as a tool to visualize and semi-quantify differences in GAG content between hydrogels. In cell-free gels, the intensity of the negatively charged contrast agent Ioxaglate varies with HA-MA (Figure 9A). For example, 0% HA-MA gels show considerably more red coloration than 2% HA-MA gels. EPIC-μCT can also give an indication of charge distribution, with more red coloration generally visible in the center regions of cell-laden constructs, where matrix synthesis was observed to be much lower (Figure 9B).

## Discussion

Hyaluronic acid (HA) is one of the most commonly studied biomaterials in cartilage tissue engineering, yet we are still discovering aspects of its significance for cartilage biology. In healthy cartilage, aggrecan is non-covalently bound to HA to create massive aggregates of fixed negative charge which provide compression resistance and swelling properties, both of which are key to its mechanical properties [22]. Many biomaterials for cartilage tissue engineering have been produced partly or wholly from HA or HA derivatives, some of which have progressed to clinical trials [13], [23], [24]. In addition, lightly crosslinked HA is an effective viscosupplementation agent for diseased or damaged joints, including those with OA [25].

In this study, a photocrosslinkable HA derivative has been shown to be a valuable addition to gelatin-based hydrogels, and specifically, has shown that the addition of HA-MA results in a substantial improvement in the developed mechanical properties, as measured using a number of different parameters. This is relevant for tissue-engineered cartilage *in vivo*, which must routinely withstand considerable stresses. After 9 weeks of culture, construct dynamic and equilibrium moduli significantly increased in a HA-MA concentration-dependent manner. After this time in culture, the majority of the compressive modulus was due to cell-secreted ECM. Additionally, inclusion of HA-MA increased failure strength relative to Gel-MA constructs, but strength was independent of HA-MA concentration over the range 0.25–2%. Although the failure strength of cell-free gels was not measured, it is likely that the cell secreted ECM is also a major factor in determining the failure strength of the cell-hydrogel constructs.

While it is clear that the mechanical properties of the constructs in this study improve with time in culture, the exact mechanism for this improvement is not clear. GAG content in articular cartilage is correlated with compressive properties [26], [27] and GAG content is the most commonly used method to measure the functional development of tissue-engineered cartilage. However the importance of total GAG content for the mechanical properties of tissue-engineered cartilage is unclear, and an important distinction was observed in this study. In contrast to the role of GAGs in cartilage, the total GAG content had very little, if any, correlation with mechanical properties of the tissue engineered constructs in this study. GAG content was relatively similar for each patient and hydrogel composition, with no significant differences in total GAG content or GAG/dry weight. When normalised within each patient, a slight increase in GAG/dry weight was measured for gels with 0.5% HA-MA, which is roughly in line with previous results from a single patient [14]. Other studies have shown that in pure HA-MA hydrogels, the correlation between mechanical properties and GAG content was also highly non-linear [13]. This must be considered when assessing the quality of tissue-engineered cartilage throughout the literature, where GAG content has been measured but not mechanical properties. Along with GAGs, the collagen network is also important for providing cartilage with mechanical strength. Total collagen content was not measured in this study, but it is likely that both the collagen content and its distribution throughout the gels contribute substantially to the mechanical properties. The hydroxyproline assay is the most common technique to measure total collagen content, but this is not practical for gels containing such a large amount of gelatin.

Cell viability was high after 28 days, indicating that the crosslinking process is well tolerated and that cells receive sufficient nourishment by diffusion through the gel. The crosslinking process has been previously shown to yield high cell viabilities [28], [29], but no study has definitely shown that no adverse cell outcomes occur. Meanwhile, photoinitiators that are active in the visible region of the spectrum are being investigated to further remove the potential for UV or radical induced cell damage [30].

Immunofluorescence analysis showed that aggrecan distribution was enhanced in gels with HA-MA, particularly towards the outer edges, and in the 1% HA-MA group, in which an almost continuous aggrecan staining can be observed. This enhanced ECM distribution may be responsible for the increases in mechanical properties when HA-MA was included. Less staining was consistently observed in the centre of the constructs, and this is most likely a consequence of lower nutrient concentrations due to diffusion gradients within the gels. Constructs with 2% HA-MA appeared to inhibit the diffusion of OCT into the tissue; this adversely affected the quality of the sections from gels with 2% HA-MA, and as such, limited emphasis should be placed on images from these gels. Cell viability, however, was not reduced in the centre regions of all tested gels, perhaps unsurprisingly, since chondrocytes habitually withstand hypoxic conditions in native cartilage. Mechanical stimulation using bioreactors or perfused medium culture systems could be used to increase mass transfer throughout these constructs, potentially further improving mechanical properties [13]. Mechanical stimulation may also simultaneously enhance chondrogenesis and matrix synthesis by mimicking the natural strains that are applied to cartilage [18], and further studies should investigate bioreactor systems to progress these materials towards regenerative medicine applications.

Collagen type X was observed in all hydrogels in this study, which could potentially be a consequence of prolonged TGF-β3 exposure or the presence of reactive oxygen species during crosslinking. Other studies have indicated that reactive oxygen species may promote hypertrophy and collagen type X production [31], [32], thereby reducing the quality of the resulting tissue. Importantly, using alternative chemical groups for photocrosslinking can reduce hypertrophy and collagen type X production. Thiol groups, for example, can quench reactive oxygen species during crosslinking, whereas acrylate-based chemistries cannot [32]. HA can be modified with thiol groups using simple procedures [33] or purchased, and could be used instead of HA-MA [34]. Such a study would also provide insight into the importance of network topology on the developed mechanical properties and cell responses [32], since a substantial proportion of the crosslinks would be expected to occur between HA and Gel-MA macromers. In addition, oxygen inhibition results in a several-minute delay in the onset of crosslinking of acrylate (and methacrylamide) systems [10], [34]. By using thiolated HA this delay is effectively removed, which would greatly reduce the required UV exposure [32] and make the process safer.

Biological variation, or donor-to-donor variation, presents a significant challenge for biomedical research. Previously, it has been identified that HA-MA can improve the stiffness of cultured Gel-MA/HA-MA constructs, but these results were obtained using chondrocytes from a single patient [14], and thus uncertainty remained over how applicable the findings were to cells from other patients. In the present study, we used chondrocytes from four patients to measure the importance of donor-to-donor variation. The similar response of each patient to these materials confirms that material composition has a significant influence on construct maturation. It is reasonable to expect that the differences observed here using *in vitro* methods would also translate to substantial differences in an *in vivo* setting, but this remains to be verified.

Cell source is an important consideration for cartilage repair, and a number of different clinically-driven routes have been explored. Therefore, this study also provides further validation for the functionality of chondrocytes from elderly patients and OA joints. Initially, expanded autologous chondrocytes were used for cartilage repair [35], but since then allogeneic chondrocytes [36] and mesenchymal stem cells [37] have been investigated. Cells from young donors are thought to be more biologically active and have greater potential for regeneration, and thus cartilage from juvenile donors has been preferred for a larger group of clinical treatments [38]. Although we have no comparative data from juvenile patients, in this study all cells were isolated from elderly patients with OA, and the cells from all patients were able to secrete ECM that significantly increased the construct stiffness. This reinforces the potential for using autologous chondrocytes even in elderly patients in combination with biomimetic hydrogels.

The hydrogels formed in this study are crosslinked using two very similar chemistries. The gelatin component is modified with methacrylamide groups, while HA is modified with methacrylate groups. Both groups are crosslinked via the reaction of unsaturated vinyl bonds however the reactivity of methacrylamide is reduced by resonance stabilisation with the adjacent nitrogen. Theoretically, methacrylamide can crosslink with methacrylate, and this has recently been demonstrated using Gel-MA and methacrylate functionalised polycaprolactone [39]. Should no preference exist, Gel-MA – HA-MA gels would be randomly inter-crosslinked on a molecular level. However, it is possible that the methacrylate groups preferentially crosslink with other methacrylate groups, or that due to their higher reactivity, methacrylate groups are favoured, particularly during the early stages of crosslinking. In this case, the network would resemble a double-network hydrogel, in which the first network is formed by crosslinked HA-MA, and the second network is crosslinked Gel-MA.

The degradation characteristics lend some weight to the hypothesis that two distinct networks may exist. For example, if the gelatin component of a gel with a composition of 9.5% Gel-MA, 0.5% HA-MA is removed by papain or proteinase K digestion, an intact, stable network of crosslinked HA-MA remains. The existence of a stable HA-MA network, even when HA-MA accounts for only one twentieth of the total dry mass indicates that the HA-MA network exists independently of the Gel-MA network. Of course this is also facilitated by the high molecular weight of the HA used here, increasing the likelihood and occurrence of HA chains overlapping.

The extent of mixing between Gel-MA and HA-MA is temperature dependent. Mixtures of these polymers have a degree of turbidity, which increases as temperature is reduced, probably as a result of the thermal gelation response of Gel-MA (Figure S1). This suggests that the network structure of crosslinked Gel-MA – HA-MA hydrogels may be temperature dependent, and possibilities may exist to further improve mechanical property development by adjusting the crosslinking temperature.

An important question also remains about the functionality of HA-MA compared to HA, and the mechanism by which HA-MA improves the mechanical properties. In cartilage, for instance, the non-covalent interaction between aggrecan and HA is stabilised by link protein [40], and HA turnover is mediated by enzymatic degradation by hyaluronidase and replacement with high molecular weight HA [41]. Preliminary data from our research (not shown) indicates that HA-MA is much less readily degraded by hyaluronidase than HA, and that link protein does not strongly bind to or recognise HA-MA. The reduced susceptibility of HA-MA to hyaluronidase has been noted in other studies, and appears, as one might expect, to depend on the degree of functionalisation [42]. Thus if HA-MA has altered functionality to HA, the mechanisms by which HA-MA improves the matrix organisation and function when added to Gel-MA gels requires further research.

There is a strong need for improved materials for cartilage tissue engineering, and biomimetic materials are a promising strategy to deliver these improvements. Currently, fibrin glue is one of the most commonly used gels to deliver cells to cartilage defects [43]. This usage is based on the familiarity of fibrin glue to orthopaedic surgeons and existing regulatory approval, rather than solid evidence demonstrating the suitability of fibrin glue for cartilage repair. Individually, Gel-MA and HA-MA are both interesting materials for tissue engineering [2], [15], [44], and here and elsewhere, mixtures of these two materials have been identified as having particularly intriguing properties [10], [14], [45]. The crosslinked hydrogels appear to be quite stable in PBS, with cell-free gels of all compositions maintaining their shape and structure for at least six months at room temperature (data not shown). The gels are susceptible to enzymatic degradation, which can be manipulated by varying the relative amount of HA-MA [45] or incorporating polymers that are not susceptible to enzymatic degradation, such as PEG [46]. Further research should consider the mechanisms by which HA-MA exerts an impact on developed mechanical properties, possibilities to enhance diffusion throughout the gel, and direct comparisons between these gels and existing clinically used materials in large preclinical animal models.

## Conclusion

In summary, combinations of Gel-MA and HA-MA are promising candidates for cartilage tissue engineering. Encapsulated chondrocytes display a predominantly rounded morphology, and secrete extracellular matrix that increases the compressive modulus by up to three-fold over 8 weeks culture. Importantly, different patients respond similarly to HA-MA, and cells from all patients were capable of substantially increasing the stiffness of Gel-MA – HA-MA hydrogels.

## Supporting Information

Figure S1 **Photographs of gel precursor solutions at 37°C and 4°C.** Mixing of Gel-MA and HA-MA is temperature dependent, with HA-MA causing much greater opacity when the mixtures are cool.
(TIF)

Figure S2 **Proton nuclear magnetic resonance spectra of gelatin, hyaluronic acid, and their photocrosslinkable derivatives.** The appearance of two peaks in the region 5.5–6.5 ppm demonstrates the addition of unsaturated, photocrosslinkable groups.
(TIF)

Figure S3 **High magnification confocal micrographs of actin structures from a 0% HA-MA construct cultured for 28 days.** Images were taken from the surface of the gel (A and C) or the centre (B). Scalebars represent 100 µm (A), 50 µm (B) or 10 µm (C).
(TIF)

Dataset S1 **Raw data from hydrogel constructs.** Data on wet weight, dry weight, compressive modulus, DNA content, and GAG content are provided in a spreadsheet for all analysed hydrogel constructs. Additionally, raw data from mechanical compression tests (time, displacement, and load) are provided.
(ZIP)
