# Supplementary figures and images for: Hyaluronic Acid Enhances the Mechanical Properties of Tissue-Engineered Cartilage Constructs
Source: PLoS One. 2014 Dec 1;9(12):e113216. doi: 10.1371/journal.pone.0113216 (PMC4249877; doi:10.1371/journal.pone.0113216)

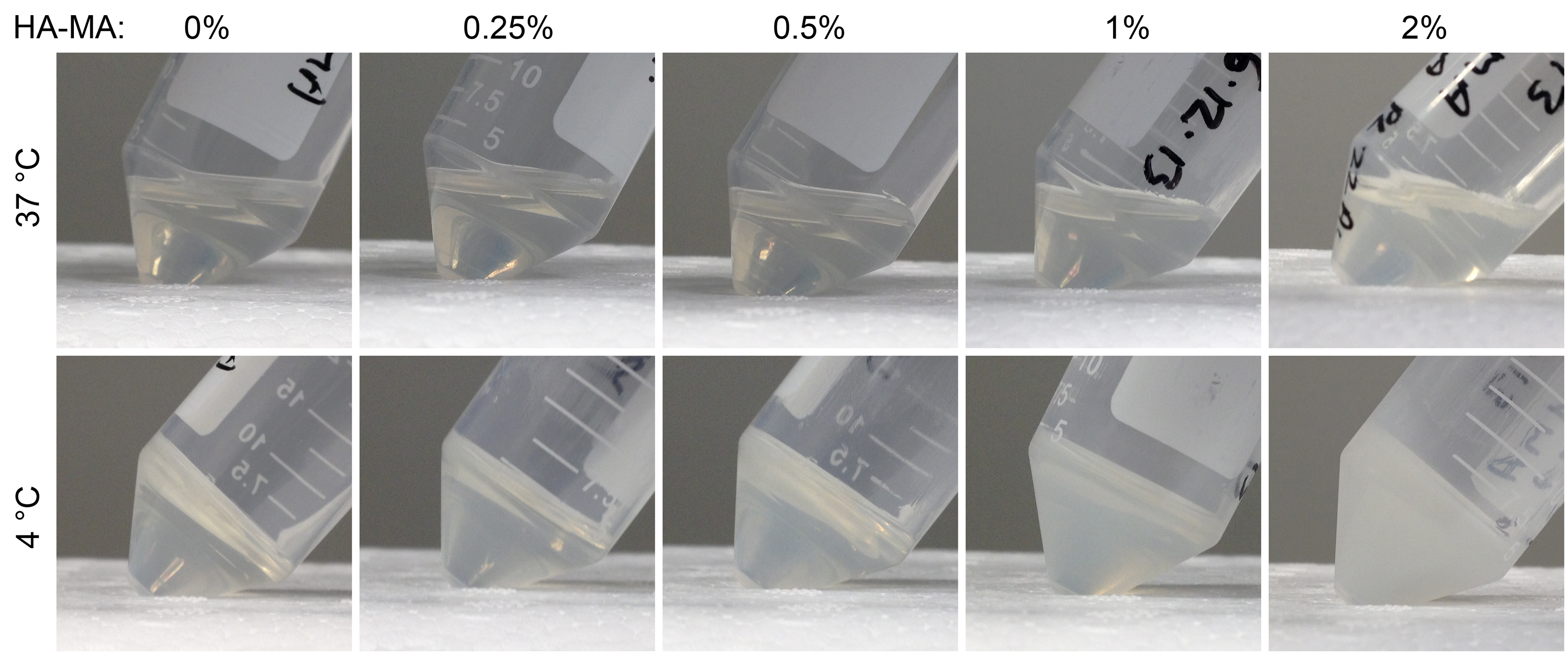

Supplement: Figure S1 — Photographs of gel precursor solutions at 37°C and 4°C. Mixing of Gel-MA and HA-MA is temperature dependent, with HA-MA causing much greater opacity when the mixtures are cool. (TIF) [file pone.0113216.s001.tif]

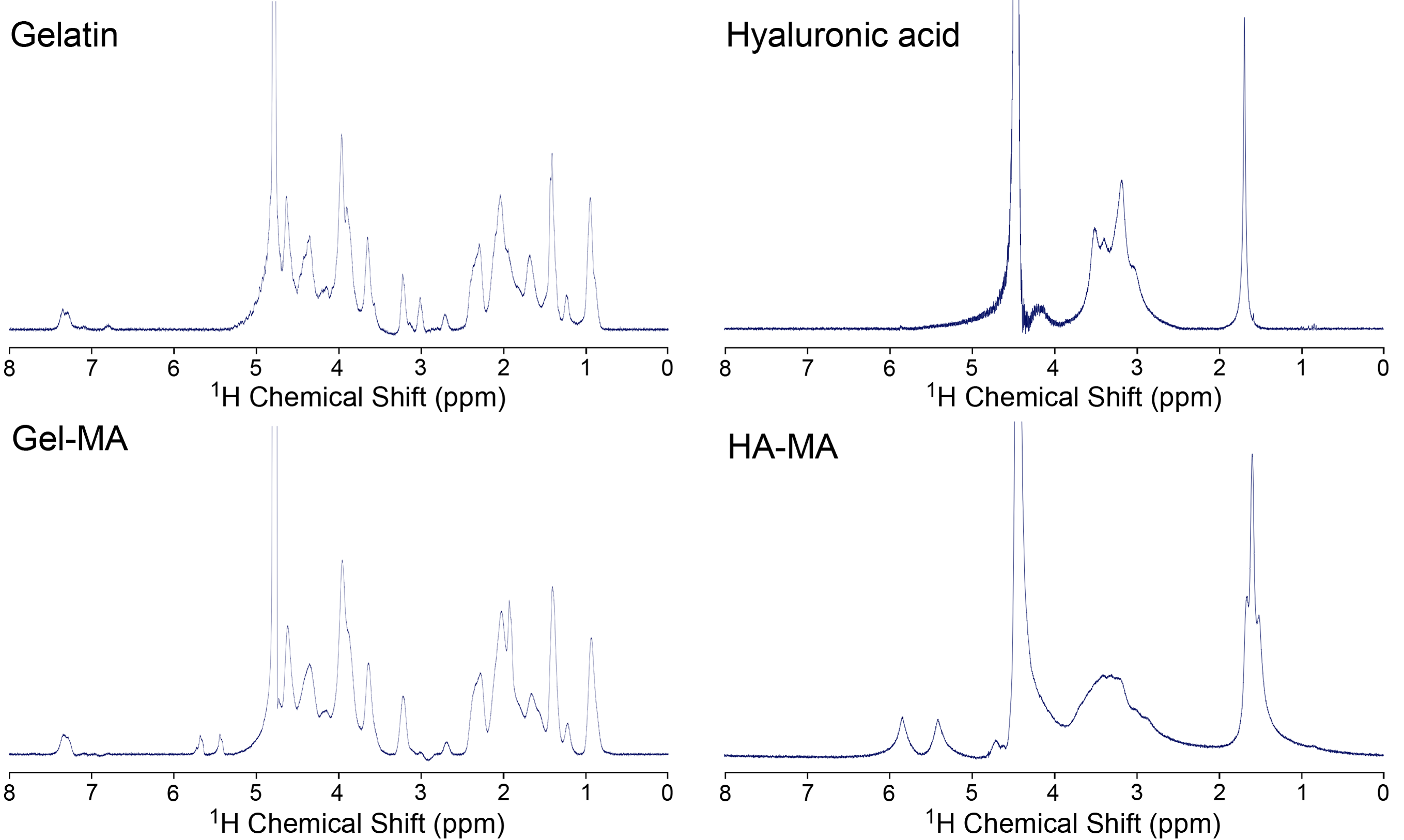

Supplement: Figure S2 — Proton nuclear magnetic resonance spectra of gelatin, hyaluronic acid, and their photocrosslinkable derivatives. The appearance of two peaks in the region 5.5–6.5 ppm demonstrates the addition of unsaturated, photocrosslinkable groups. (TIF) [file pone.0113216.s002.tif]

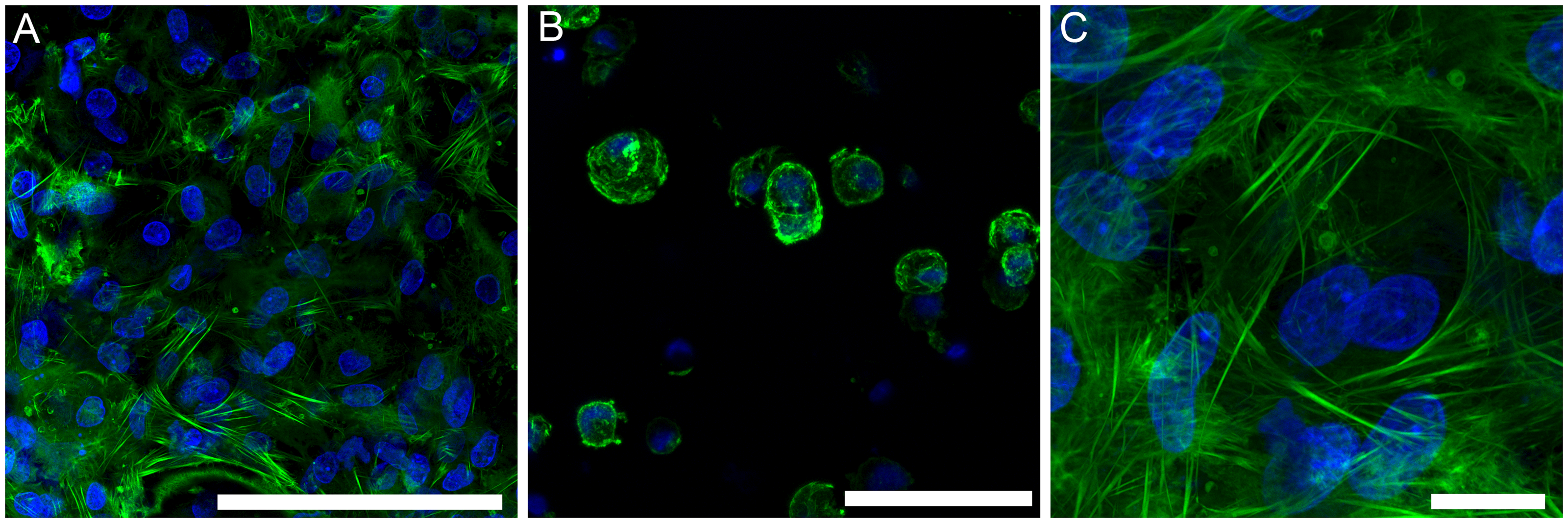

Supplement: Figure S3 — High magnification confocal micrographs of actin structures from a 0% HA-MA construct cultured for 28 days. Images were taken from the surface of the gel (A and C) or the centre (B). Scalebars represent 100 µm (A), 50 µm (B) or 10 µm (C). (TIF) [file pone.0113216.s003.tif]
